# The Hausa 12-item short-form health survey (SF-12): Translation, cross-cultural adaptation and validation in mixed urban and rural Nigerian populations with chronic low back pain

**DOI:** 10.1371/journal.pone.0232223

**Published:** 2020-05-07

**Authors:** Aminu Alhassan Ibrahim, Mukadas Oyeniran Akindele, Sokunbi Oluwaleke Ganiyu, Bashir Kaka, Bashir Bello Abdullahi, Surajo Kamilu Sulaiman, Francis Fatoye

**Affiliations:** 1 Department of Physiotherapy, Faculty of Allied Health Sciences, College of Health Sciences, Bayero University Kano, Kano, Kano State, Nigeria; 2 Department of Physiotherapy, Muhammad Abdullahi Wase Teaching Hospital, Hospital Management Board, Kano, Kano State, Nigeria; 3 Department of Physiotherapy, Sir Muhammadu Sunusi Specialist Hospital, Hospital Management Board, Kano, Kano State, Nigeria; 4 Department of Physiotherapy, Faculty of Allied Health Sciences, College of Medicine, Kaduna State University, Kaduna, Nigeria; 5 Department of Health Professions, Faculty of Health, Psychology and Social Care, Manchester Metropolitan University, Manchester, United Kingdom; Iranian Institute for Health Sciences Research, ISLAMIC REPUBLIC OF IRAN

## Abstract

**Introduction:**

Measuring health-related quality of life (HRQOL) in patients with chronic low back pain (LBP) is crucial to monitor and improve the patients’ health status through effective rehabilitation. While the 12-item short-form health survey (SF-12) was developed as a shorter alternative to the 36-item short-form health survey for assessing HRQOL in large-scale studies, to date, no cross-culturally adapted and validated Hausa version exists. This study aimed to translate and cross-culturally adapt the SF-12 into Hausa language, and test its psychometric properties in mixed urban and rural Nigerian populations with chronic LBP.

**Methods:**

The Hausa version of the SF-12 was developed following the guidelines of the International Quality of Life Assessment project. Fifteen patients with chronic LBP recruited from urban and rural communities of Nigeria pre-tested the Hausa SF-12. A consecutive sample of 200 patients with chronic LBP recruited from urban and rural clinics of Nigeria completed the instrument, among which 100 respondents re-tested the instrument after two weeks. Factorial structure and invariance were assessed using confirmatory factor analysis (CFA) and multi-group CFA respectively. Multi-trait scaling analysis (for convergent and divergent validity) and known-groups validity were performed to assess construct validity. Composite reliability (CR), internal consistency (Cronbach’s α), intraclass correlation coefficients (ICC), and Bland–Altman plots were computed to assess reliability.

**Results:**

After the CFA of the original conceptual SF-12 model, 2 redundant items were removed and 4 error terms were allowed to covary, thus providing adequate fit to the sample. The refined model demonstrated good fit and evidence of factorial invariance in three demographic groups (age, gender, and habitation). Convergent (11:12; 91% success rate) and divergent (10:12; 83% success rate) validity were satisfactory. Known-groups comparison showed that the instrument discriminated well for those who differed in age (p < 0.05) but in gender and habitation (p > 0.05). The physical component summary and the mental component summary demonstrated acceptable CR (0.69 and 0.79 respectively), internal consistency (α = 0.73 and 0.78 respectively), test-rest reliability (ICC = 0.79 and 0.85 respectively), and good agreement between test-retest values.

**Conclusions:**

The Hausa SF-12 was successfully developed and showed evidence of factorial invariance across age, gender, and habitation. The instrument demonstrated satisfactory construct validity, internal consistency, and test-retest reliability. However, stronger psychometric properties need to be established in general population and other patients groups in future studies. The instrument can be used clinically and for research in Hausa-speaking patients with chronic LBP.

## Introduction

Low back pain (LBP) constitutes a significant problem of the contemporary society as it affects people all age groups [1]. It is now considered as the leading cause of disability globally than any other condition [2]. LBP is not only a major source of incapacity but also absenteeism from work and lost productivity [3,4], hence it imposes considerable health and economic cost on individuals, families, and society [5–7]. Though most episodes of acute LBP tend to have a favorable prognosis, recurrences within a year are common [8] and about 20% develop chronic LBP [4].

Chronic LBP is an important clinical and public health problem as sufferers may continue to experience pain and functional disability which often interfere with daily life activities and subsequently reduce quality of life [9]. The association between intensity of back pain and quality of life in patients with chronic LBP has been demonstrated in several cross-sectional and prospective studies [9–12]. Consequently, the goals of treatment for chronic LBP disorder often focus on improving the functional status and quality of life of the patients [13]. Thus, health-related quality of life (HRQOL) is an important outcome and its measurement is therefore imperative for clinicians to monitor and improve patients’ health status through effective rehabilitation. However, this necessitates use of psychometrically sound instruments to evaluate HRQOL [14].

The medical outcomes study 36-item short-form health survey (SF-36) is perhaps the most widely used instrument to assess perceived health status. Since its development, it has been used as a generic instrument to evaluate or monitor HRQOL in the general population and people with different chronic illnesses including LBP [15–18]. However, owing to its administrative burden, the 12-item short-form health survey (SF-12) was developed as an alternative to the SF-36 for use in large-scale studies to assess overall physical and mental health outcomes [19]. The SF-12 has the advantage of being easier and quicker to complete [17], thus minimizing the costs for data collection and management [20].

The SF-12 consists of 12 items taken from the eight subscales of the SF-36. Similar to the SF-36, it assesses two global health constructs viz the physical component summary (PCS) and the mental component summary (MCS) [19]. The SF-12 has been found to be highly correlated with SF-36 in terms of the PCS and MCS [19]. Importantly, the questionnaire proved to be valid and reliable in assessing overall health status among the general population in many different countries [20]. More specifically, it has been shown to be an adequate measure of HRQOL in different patient groups such as LBP [21], osteoarthritis and rheumatoid arthritis [22], ankylosing spondylitis [23], retinal diseases [24], obesity [25], and mental health disorders [26].

The adaptation of health status measures for use in other than the source language is essential since it does not only permit the collection of valid and reliable data but also minimize the exclusion of subjects who cannot speak the source language [27,28]. However, the adaptation of a health status self-administered instrument for use in a new culture/language must follow methodological standards that ensure equivalency between the source and target versions of the instrument [28–30]. While the SF-12 has been successfully adapted into many different languages/cultures [31–36], to date, no cross-culturally adapted and validated Hausa version exists. Given that Hausa is a widely spoken language not only in Nigeria but also in most West African societies [37], adapted Hausa version of the SF-12 is believed to enhance accessibility and utilization of the tool for evaluating health status in Hausa-speaking population.

This study aimed to translate and cross-culturally adapt the SF-12 into Hausa language, and test its psychometric properties in mixed urban and rural Nigerian populations with chronic LBP.

## Material and methods

### Ethical considerations

This study was approved by the Health Research Ethics Committee, Ministry of Health Kano State (Ref: MOH/Off/797/T.I./651). Written informed consent was obtained from all participants prior to participating in the study.

### Study designs

Translation, cross-cultural adaptation and cross-sectional study of psychometric properties.

### The 12-item short-form health survey (SF-12)

The SF-12 consists of 12-items and 8 subdomains: physical functioning (PF), role-physical (RP), bodily pain (BP), general health (GH), vitality (VT), social functioning (SF), role-emotional (RE), and mental health (MH). The subscales PF, RP, BP, and GH forms the physical component summary (PCS-12) scores whereas the subscales VT, SF, RE, and MH forms the mental component summary (MCS-12) scores. Each item of the questionnaire has response categories which vary from 2- to 6-point scales and raw scores for items ranging from 1 to 6. The raw scores are summated and linearly transformed into 0–100 scale [38] with a higher score indicating better health status. We used a web-based scoring tool (www.orthotoolkit.com/sf-12/) to compute the PCS-12 and MCS-12 scores.

### Translation and cross-cultural adaptation

Written permission to translate the SF-12 health survey into Hausa language was obtained from the original developer. The cross-cultural adaptation followed the guidelines recommended by the International Quality of Life Assessment (IQOLA) project [39].

Two bilingual (English and Hausa) translators independently forward-translated the English SF-12 into Hausa. The first translator was a professional linguist and unaware of the concepts of the questionnaire. The second translator was a clinical physiotherapist with ample experience in questionnaire translation and aware of the concepts being examined. The aim of reaching conceptual equivalence with the original source version rather than literal equivalence while reflecting the lay language used in Hausa culture regardless of age and educational level was emphasized to both the translators. This stage led to the production of two forward Hausa SF-12 versions which were then reconciled and synthesized into one version following discussion and consensus among the forward translators with coordination of the primary author. The synthesized version was then translated back into English by two, independent professional translators who had no medical background and access to the original version of the questionnaire. This ensured that the translated questionnaire was reflecting the meaning in the original questionnaire (content validity).

To evaluate face validity, an expert review committee consisting of all the translators, the primary author and an academic physiotherapist with proficiency in methodology met and produced a pre-final version of the Hausa SF-12 after reaching consensus. The pre-final version was then pilot-tested in 15 patients with chronic LBP recruited from both urban and rural communities to assess comprehensibility and applicability. Upon completion of the questionnaire, cognitive debriefing (i.e. verbal pre-testing) was carried out by asking the participants to comments on the questionnaire items and their perceived meaning of chosen responses. The primary author with the consultation of the translators and methodologist then reviewed the questionnaire for problematic items, responses, statements, phrases, and words in terms of clarity and acceptability. This ensured that the original meaning was not lost or altered while reaching cultural equivalence. Finally, a professional translator independently proofread the final Hausa SF-12 translation for any minor errors that may have been missed during the translation and cultural adaptation process. This led to the production of the final version of the Hausa SF-12 (see S1 Appendix).

### Psychometric testing

The procedure used throughout this section has been used in the cross-cultural adaptation of other Hausa self-report measures as described elsewhere [40].

#### Sample size estimation

The “Quality criteria for measurement properties of health status questionnaires” suggest that a sample size of ≥ 50 would be sufficient for reliability, construct validity, and ceiling/floor effects analyses whereas 4–10 subjects per variable (Rules-of-thumb) would be sufficient for factorial structure analysis [41]. Based on these suggestions, we believed that recruiting 200 participants would be adequate to test the psychometric properties of the Hausa SF-12.

#### Participants

The participants were consecutive LBP patients presenting to the out-patient clinics of Murtala Muhammad Specialist Hospital, Dawakin-Kudu General Hospital, Wudil General Hospital, and Kura General Hospital all in Kano State, Northwestern Nigeria, between February and May 2018. Urban patients were recruited from the specialist hospital while rural patients were recruited from the general hospitals. Both urban and rural patients were recruited to have broader applicability of the instrument in both urban and rural areas, as well as across all levels of literacy or illiteracy. Participants were included if they were both sexes, aged 18–70 years, suffering from LBP for 12 weeks or greater, and fluent in Hausa language. They were excluded if their LBP was due to serious spine pathology (e.g. infection, malignancy, fracture, osteoporosis or inflammatory disease), cognitive impairment or impaired capacity to be interviewed, and pregnancy.

#### Procedure for data collection

*Training of assessors*. Physiotherapists in the respective hospitals received training on interviewer-administration of measures as many patients in Nigeria especially rural dwellers are not literate (ability to read and write in Hausa). This was deemed necessary to minimize survey error even though majority of the physiotherapists are familiar with the questionnaire. All the physiotherapists were staff of Hospital Management Board Kano State, Nigeria, with clinical experience between two to five years. The therapists received a one-day training session based on verbal pretesting of measures. The session included face-to-face and group-based training coordinated by the primary researcher in a classroom.

*Data collection*. Information on demographic characteristics (age, gender, marital status, education level, occupation, and habitation) and clinical data (duration of pain) were obtained and recorded for each participant. The Hausa SF-12 was interviewer-administered. However, literate participants’ were self-administered where necessary. The questionnaire was re-administered at an interval of approximately 14 days after the first measurement to minimize participants recalling previous answers.

### Statistical analysis

All statistical analyses were performed using IBM SPSS for Windows version 24.0 (IBM Corp, Armonk, NY) at an alpha level of 0.05 except for confirmatory factor analysis (CFA) which was performed with IBM AMOS software, version 26.0 for Windows. Descriptive statistics of mean, standard deviation (SD), frequencies, and percentages were used to summarize the data. Visual (normal distribution curve and Q-Q plot) and statistical methods (Kolmogorov-Smirnov and Shapiro-Wilk’s test) were used to test the normality of the data.

#### Floor and ceiling effects

Floor or ceiling effects are considered to exist if more than 15% of respondents scored the minimum or maximum possible score [41]. Potential floor or ceiling effects of the Hausa SF-12 were investigated by calculating the percentage of respondents indicating the minimum or maximum possible score in all the items and the two components summary measures.

#### Factorial structure

Factor structure of the Hausa SF-12 was investigated by first performing exploratory factor analysis (EFA) using principal component analysis with varimax rotation to verify the original conceptual SF-12 model. It was hypothesized that a two-factor model (reflecting PCS-12 and MCS-12) would be obtained with eigenvalues greater than 1 [33,34]. Confirmatory factor analysis (CFA) using maximum likelihood estimates was then performed with the two-factor model to verify adequate fit to our data. Modification indices were used to improve model fit by verifying item’s redundancy or those with low factor loadings, and correlation between the items [42]. Goodness-of-fit indicators to the data variance/covariance matrix were assessed with the ratio of chi-square to degrees of freedom (χ2/df), comparative fit index (CFI), Tucker-Lewis index (TLI), standardized root mean square residual (SRMR), and root mean square error of approximation (RMSEA) [43]. Multiple fit statistics were chosen as χ2 alone, even though being regarded as the traditional measure of model fit, is very sensitive to sample size [44]. According to conventional criteria, an acceptable model fit would be indicated by χ2/df ≤ 2.0, CFI ≥ 0.95, TLI ≥ 0.90, SRMR ≤ 0.08, and RMSEA ≤ 0.06 [43, 45]. Additionally, average variance extracted (AVE) and composite reliability (CR) for each model were computed. An AVE value ≥ 0.5 indicates acceptable convergent validity while CR value ≥ 0.7 indicates acceptable reliability [46]. However, if AVE is < 0.5, but CR is > 0.6, the convergent validity is still acceptable [46].

#### Factorial invariance

Factorial invariance or measurement invariance was investigated by performing multi-group CFA across age (younger adults: 18–44 years, and adults: >45 years), gender (men and women), and habitation (urban and rural) groups. We categorized age into two groups only as further categorization would lead to severely unbalanced groups (due to small sample size) which might affect the results [47]. Factorial invariance assesses the psychometric equivalence of a construct across groups [48]. Factorial invariance of the Hausa SF-12 was assessed by evaluating the following levels of invariance: a) configural invariance, an unconstrained model testing for the model fit of baseline model across groups, b) metric invariance, a constrained model testing factor loadings equivalence across groups (weak invariance), and c) scalar invariance, a constrained model reflecting factor loadings and item intercepts across groups [49,50]. The configural model serves as the baseline against which all subsequent invariance models were compared [42]. Invariance of the models was tested using likelihood ratio test with chi-square difference (Δχ2) statistics and change in alternative fit indices with ΔCFI, ΔRMSEA, and ΔSRMR. Invariant model was considered when Δχ2 is non-significant (p > 0.05), χ2/df ≤ 2.0, ΔCFI > –0.01, ΔRMSEA < 0.015, and ΔSRMR < 0.03 [43, 51, 52].

#### Construct validity

Construct validity was investigated by assessing convergent, divergent, and known-groups validity. Convergent and divergent validity were assessed using multi-trait scaling analysis with the use of Pearson’s correlation coefficients (normally distributed data). Pearson’s correlation coefficients (*r*) were interpreted as being strong (> 0.6), moderate (0.3–0.6), and weak/low (< 0.3) [53].

For convergent validity, it was expected that item scores would correlate higher with own hypothesized component (Pearson’s *r* > 0.4) than other component [34,36]. Therefore, items 1,2, 3,4,5, and 8 scores would correlate more with the PCS-12 scores whereas items 6,7,9,10,11, and 12 scores would correlate more with the MCS-12 scores. For divergent validity, those items with less in common would demonstrate lower correlations (Pearson’s *r* < 0.4) [36]. Additionally, the PCS-12 and MCS-12 scores were expected to correlate weakly (Pearson’s *r* < 0.4) since they measure a different latent concept [54].

Known-groups validity (the ability of an instrument to discriminate between extreme groups) was assessed by comparing mean scores of scales and components by age, gender, and habitation using one-way analysis of variance (ANOVA) or independent t-test. Effect sizes were interpreted according to Cohen’s d as either trivial (< 0.2), small (≥ 0.2 and < 0.5), moderate (≥ 0.5 and < 0.8) or large (≥ 0.8) [55]. We hypothesized that older subjects, women, and rural subjects would report poorer health [33,56,57].

#### Internal consistency

Internal consistency for the PCS-12 and MCS-12 was assessed using Cronbach’s alpha (α). A Cronbach-α value of ≥ 0.70 is generally regarded as acceptable [41].

#### Test-retest reliability

Test-retest reliability of the PCS-12 and MCS-12 was assessed by calculating intraclass correlation coefficient (ICC) for agreement using a two-way random effects ANOVA model (which assumes that measurement errors could arise from either raters or subjects). Confidence intervals (CI) were also computed for the ICC. A coefficient ≥ 0.70 was considered adequate for test-retest reliability [41]. Additionally, limits of agreement were assessed with Bland–Altman plots [58]. The Bland–Altman plots were used to visually assess the level of agreement between test-retest measurements by plotting mean PCS-12 and MCS-12 scores against difference in PCS-12 and MCS-12 scores respectively.

## Results

### Translation and cross-cultural adaptation

The translation of the Hausa SF-12 was easy as there were no major translation problems encountered except for items 2 and 11. In item 2, the phrase “pushing a vacuum cleaner and bowling” was modified to “lifting a dustbin and archery”. In the Hausa culture, pushing a vacuum cleaner is not familiar, hence the phrase “lifting a dustbin” was used. Similarly, bowling which refers to a target sport and recreational activity that involves rolling or throwing a heavy ball towards a target is not commonly practiced in Hausa culture. In contrast, archery which involves a skill of using a bow to shoot arrows is commonly practiced in the Hausa culture and can be an alternative to bowling. Furthermore, in item 11, the phrase “depressed and heart-broken” was used in place of the phrase “down-hearted and blue” as this phrase has no equivalence in the Hausa culture. The translators ensured that the original meaning is not lost or altered while attaining cultural equivalence between the original source version and the Hausa version. Results of the pilot testing suggest that all the items were clear and comprehensive.

### Psychometric testing

#### Participants

All the respondents completed the instrument signifying 100% response rate. There were 123 (61.5%) males and 77 (38.5%) females. Their mean age was 45.5±14.5 years with majority of them living in rural areas (60%). Slightly over half of the respondents were Hausa non-literates (55.5%) and self-employed farmers and traders (56.0%). The demographic characteristics of the respondents are fully shown in Table 1.

**Table 1:** Demographic characteristics of the respondents.

Variables	N = 200
Age, years, mean ± SD	45.5 ± 14.5
Gender, *n (%)*, male: female	123 (61.5), 77 (38.5)
Habitation, *n (%)*, urban: rural	80 (40.0), 120 (60.0)
Tribe, *n (%)*, Hausa: others	199 (99.0), 1 (1.0)
Marital status, *n (%)*, married: unmarried	157 (78.5), 43 (21.5)
Educational status, *n (%)*	
None	66 (33.0)
Completed primary education	30 (15.0)
Completed secondary education	41 (20.0)
Completed tertiary education	63 (31.5)
Literacy (ability to read and write), *n (%)*	
Literate	89 (44.5)
Non-literate	111 (55.5)
Occupational status, *n (%)*	
Paid work (government or private)	49 (24.5)
Self-employed (farming/trading)	112 (56.0)
Student	17 (8.5)
Unemployed	16 (8.0)
Retiree	6 (3.0)

SD, standard deviation

#### Missing data, floor or ceiling effects

All the 200 respondents completed the Hausa SF-12 without missing values. The mean score for the PCS-12 was 34.5 (SD = 6.94), and 38.9 (SD = 10.1) for the MCS-12. Floor effects were found in items 2–7 (PF, RP and RE scales) whereas ceiling effects were found in items 1 (GH scale), 5 (RP2 scale), 7 (RE2 scale), and 10 (VT scale) (Table 2).

**Table 2:** General characteristics of the Hausa SF-12 (N = 200).

Hausa SF-12			Mean row scores (SD)		Item response frequency (n)							Floor effects n (%)	Ceiling effects n (%)
Item	Question	Scale			1		2	3	4	5	6		
Q1	Health rating in general	GH	2.55 (0.92)		19		94	45	42	-	NA	19 (9.5)	42 (21.5)
Q2	Limitations in moderate physical activities	PF1	1.73 (0.52)		62		130	8	NA	NA	NA	62 (31.0)	8 (4.0)
Q3	Limitations in climbing several flights of stairs	PF2	1.68 (0.54)		72		120	8	NA	NA	NA	72 (36.0)	8 (4.0)
Q4	Accomplished less because of physical health	RP1	1.15 (0.35)		170		130	NA	NA	NA	NA	170 (85.0)	30 (15.0)
Q5	Limited in work or activities because of physical health	RP2	1.13 (0.46)		139		61	NA	NA	NA	NA	139 (69.5)	61 (30.5)
Q6	Accomplished less as a result of emotional problems	RE1	1.12 (0.33)		175		25	NA	NA	NA	NA	175 (87.5)	25 (12.5)
Q7	Not careful in work or activities as a result of emotional problems	RE2	1.41 (0.49)		118		82	NA	NA	NA	NA	118 (59.0)	82 (41.0)
Q8	How much did pain interfere with work inside and outside the home?	BP	2.71 (0.99)		28		48	82	32	4	NA	28 (14.0)	4 (2.0)
Q9	How much of the time did you feel calm and peaceful?	MH1	3.10 (1.22)		21		50	43	59	27	-	21 (10.5)	27 (13.5)
Q10	How much of the time did you have a lot of energy?	VT	3.03 (1.29)		30		39	60	36	35	-	30 (15.0)	35 (17.5)
Q11	How much of the time did you feel downhearted and blue?	MH2	3.33 (1.40)		21		51	26	45	56	1	21 (10.5)	1 (0.50
Q12	How much of the time did physical health or emotional problems interfere with social activities?	SF	3.13 (1.17)		22		41	45	73	19	NA	22 (11.0)	19 (9.5)
	**Summary component**												
	PCS-12		34.5 (6.94)									1 (0.5)	1 (0.5)
	MCS-12		38.9 (10.1)									1 (0.5)	1 (0.5)

SD, standard deviation; NA, not applicable; PF, physical functioning; RP, role-physical; BP, bodily pain; GH, general health; VT, vitality; SF, social functioning; RE, role-emotional; MH, mental health; PCS-12, physical component summary; MCS-12, mental component summary

#### Factorial structure, convergent validity, and composite reliability

**Fig 1:**
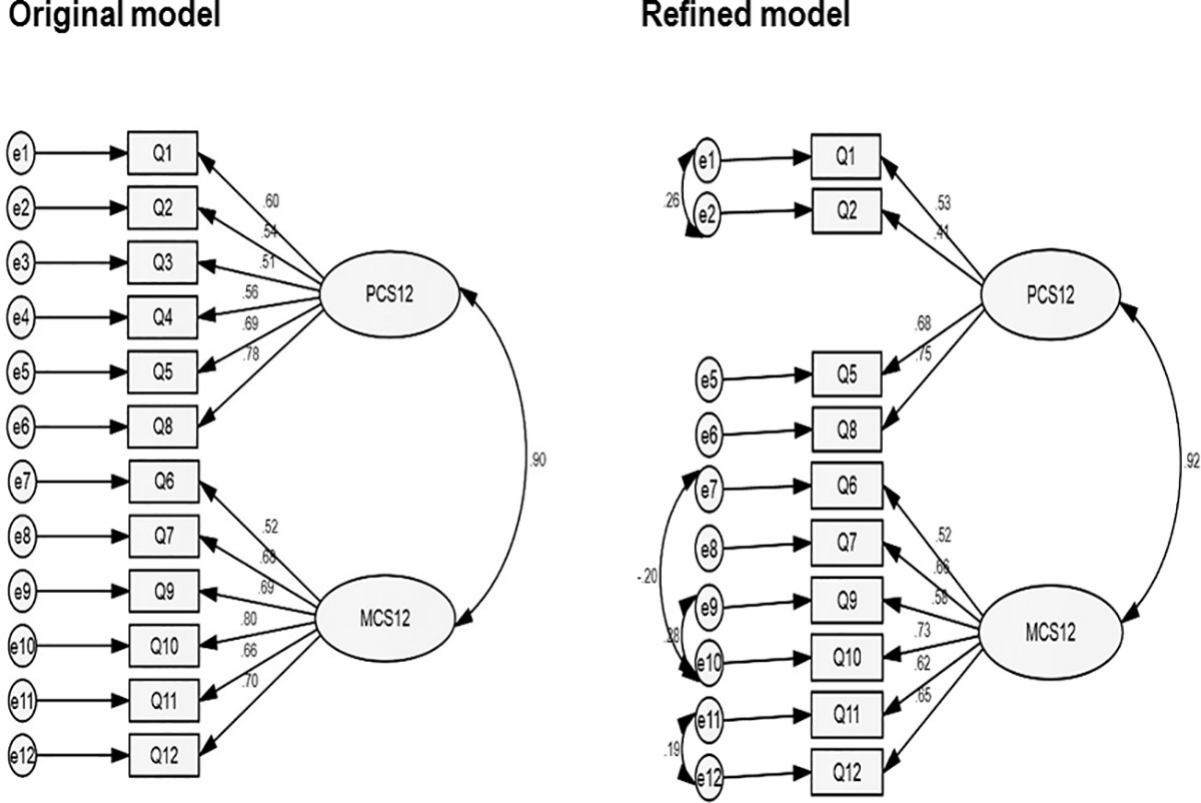
Factor structure of the Hausa SF-12. Model fit of the original conceptual SF-12 model (CFA: χ2/df = 2.5, CFI = 0.488, TLI = 0.363, SRMR = 0.091, RMSEA = 0.086, σ2 = 0,90) and the refined model fitted to the Hausa sample of patients with chronic LBP (CFA: χ2/df = 1.6, CFI = 0.970, TLI = 0.954, SRMR = 0.044, RMSEA = 0.056, σ2 = 0,92).

The two-factor conceptual model of the SF-12 was confirmed explaining 49.7% of the total variance following the EFA. Factor one included items 6, 7, 9, 10, 11, and 12, which reflect MCS-12 while factor two included items 1, 2, 3, 4, 5, and 8 which reflect PCS-12. The item-factor loadings (λ) for the PCS-12 ranged .41–.75 while that of the MCS-12 ranged .52–.73. The CFA for the original conceptual SF-12 model and refined model fitted to the Hausa sample of patients with chronic LBP is presented in Fig 1. The original conceptual SF-12 model demonstrated poor fit. However, to improve the fit of the model, items 3 (climbing several flights of stairs) and 4 (accomplished less than you would like) were removed due to redundancy. Additionally, 4 error terms were allowed to covary (e1–e2, e7–e10, e9–e10, and e11–e12). The refined model demonstrated adequate fit to the sample explaining 92% of variance (Fig 1).

Table 3 shows the CFA, AVE, and CR of the refined model fitted to different groups. The refined model demonstrated good fit in all the tested demographic groups evidenced by the adequate fit statistics and indices (χ2/df < 2.0, CFI > 0.95, TLI > 0.90, SRMR < 0.08, and RMSEA < 0.06) except for the young adult group (18–44 years) which showed fair RMSEA (0.072). However, since model fit for the overall population (refined model) was adequate, we decided to use the refined model as baseline model in subsequent analyses (i.e. factorial invariance) involving the young adult group. Similar to the refined model, the demographic groups demonstrated inadequate AVE (< 0.5) for both the PCS-12 and MCS-12 while the CR was adequate especially for the MCS-12 (> 0.7).

**Table 3:** Confirmatory factor analysis, average variance extracted, and composite reliability of the refined model fitted to different groups.

Model		Confirmatory factor analysis						AVE		CR	
	n	χ2 (df)	χ2/df	CFI	TLI	SRMR	RMSEA (95%CI)	PCS-12	MCS-12	PCS-12	MCS-12
Refined (baseline) model	200	48.5 (30)	1.61	0.970	0.954	0.044	0.056 (0.023–0.086)	0.37	0.39	0.69	0.79
Younger adult (18–44 years)	84	42.9 (30)	1.43	0.950	0.926	0.059	0.072 (0.000–0.118)	0.36	0.41	0.69	0.80
Adult (> 45 years)	116	43.9 (30)	1.46	0.958	0.938	0.053	0.064 (0.007–0.102)	0.36	0.38	0.68	0.78
Men	123	37.0 (30)	1.23	0.981	0.972	0.050	0.044 (0.000–0.085)	0.36	0.42	0.69	0.81
Women	77	42.5 (33)	1.28	0.956	0.940	0.061	0.062 (0.000–0.110)	0.37	0.41	0.69	0.80
Urban	80	32.9 (32)	1.02	0.996	0.994	0.057	0.019 (0.000–0.086)	0.38	0.39	0.70	0.79
Rural	120	42.3 (30)	1.40	0.968	0.953	0.050	0.059 (0.000–0.097)	0.33	0.44	0.66	0.83

χ2, chi-square; df, degrees of freedom; CFI, comparative fit index; TLI, Tucker-Lewis index; SRMR, standardized root mean square residual; RMSEA, root mean square error of approximation; CI, confidence intervals; AVE, average variance extracted; CR, composite reliability; PCS-12, physical component summary; MCS-12, mental component summary

#### Factorial invariance

The results of the multi-group CFA across age, gender, and habitation are presented in Table 4. The configural model for all the groups showed a good fit. The addition of constraints for equal factor loadings (metric invariance) and item intercepts (strong invariance) did not result in a significant worsening of the model fit in all the groups.

**Table 4:** Factorial invariance tests regarding age, gender, and habitation (Total sample, N = 200).

	Model fit test statistics and fit indices						Δ in model fit test statistics and fit indices			
Models	χ2(df)	χ2/df	CFI	TLI	SRMR	RMSEA (95%CI)	Δ χ2 (Δ df)	ΔCFI	ΔSRMR	ΔRMSEA
**Age (18–44 years, > 45 years)**										
Configural invariance	88.1 (60)	1.46	0.953	0.929	0.059	0.049 (0.024–0.069)	-	-	-	-
Metric invariance	96.0 (70)	1.37	0.956	0.944	0.073	0.043 (0.018–0.063)	7.9 (10)^†^	–0.003	–0.014	0.006
Scalar invariance	108.9 (80)	1.36	0.951	0.945	0.071	0.043 (0.019–0.062)	12.9 (10)^†^	0.005	0.002	0.000
**Gender (Men, Women)**										
Configural invariance	73.2 (60)	1.22	0.978	0.966	0.051	0.033 (0.003–0.057)	-	-	-	-
Metric invariance	78.4 (70)	1.12	0.986	0.982	0.053	0.025 (0.000–0.050)	5.25 (10)^†^	–0.008	–0.002	0.008
Scalar invariance	85.2 (80)	1.06	0.991	0.990	0.054	0.018 (0.000–0.045)	6.80 (10)^†^	–0.004	–0.001	0.007
**Habitation (Urban, Rural)**										
Configural invariance	81.8 (60)	1.36	0.964	0.946	0.057	0.043 (0.013–0.065)	-	-	-	-
Metric invariance	87.6 (70)	1.25	0.971	0.963	0.066	0.036 (0.000–0.057)	5.80 (10)^†^	–0.007	–0.009	0.007
Scalar invariance	91.8 (80)	1.14	0.971	0.963	0.059	0.036 (0.000–0.057)	4.20 (10)^†^	0.000	0.007	0.000

Δ, change; χ2, chi-square; df, degrees of freedom; CFI, comparative fit index; TLI, Tucker-Lewis index; SRMR, standardized root mean square residual; RMSEA, root mean square error of approximation; CI, confidence intervals

^†^*p* > 0.05

#### Construct validity

Table 5 shows the convergent and divergent validity (*n =* 200) of the Hausa SF-12. Regarding convergent validity, items pertaining to physical health correlated higher with the PCS-12 except for item 4 (RP1) (*r* < 0.4) whereas items pertaining to mental health correlated more with the MCS-12, all (11:12; 91% success rate), thus confirming the hypothesized item component correlations. For divergent validity, items belonging to the PCS-12 had the lowest correlation with the MCS-12 whereas items belonging to the MCS-12 had the lowest correlation with the PCS-12 except for item 5 (RP2) and 8 (BP) (*r* > 0.4), all (10:12; 83% success rate), thus confirming the hypothesized item component correlations. The results also showed that the PCS-12 and MCS-12 were weakly correlated (*r* = 0.18) to each other, thus indicating discriminant validity as hypothesized (Table 5).

**Table 5:** Convergent and divergent validity of the Hausa SF-12 (N = 200).

Item	Scale	Question	Correlation coefficients	
			PCS-12	MCS-12
Q2	PF1	Limitations in moderate physical activities	**0.56**	0.14
Q3	PF2	Limitations in climbing several flights of stairs	**0.50**	0.28
Q4	RP1	Accomplished less because of physical health	**0.37**	0.33
Q5	RP2	Limited in work or activities because of physical health	**0.47**	0.45
Q8	BP	How much did pain interfere with work inside and outside the home?	**0.66**	0.50
Q1	GH	Health rating in general	**0.70**	0.29
Q12	SF	How much of the time did physical health or emotional problems interfere with social activities?	0.35	**0.61**
Q6	RE1	Accomplished less as a result of emotional problems	0.09	**0.53**
Q7	RE2	Not careful in work or activities as a result of emotional problems	0.25	**0.60**
Q10	VT	How much of the time did you have a lot of energy?	0.37	**0.66**
Q9	MH1	How much of the time did you feel calm and peaceful?	0.20	**0.66**
Q11	MH2	How much of the time did you feel downhearted and blue?	0.24	**0.65**
**Component**				
PCS-12			1.00	**0.68**
MCS-12			**0.18**	1.00
**Summary**				
**Convergent validity**				
Range of correlation			0.37–0.70	0.61–0.68
Success rate (%)			91	
**Divergent validity**				
Range of correlation			0.20–0.37	0.14–0.50
Success rate (%)			83	

PF, physical functioning; RP, role-physical; BP, bodily pain; GH, general health; VT, vitality; SF, social functioning; RE, role-emotional; MH, mental health; PCS-12, physical component summary; MCS-12, mental component summary

Known-groups validity of the Hausa SF-12 regarding age group shows significant differences in the RP, BP, GH, VT, and MH scales, as well as PCS-12 scores (p < 0.05) with small to moderate effect size (Table 6). The youngest age group (18–24 years) exhibited higher mean scales and components scores. A decline in mean scores with a higher age group was generally observed across the different scales and components. In contrast, Table 7 shows no significant gender or habitant differences in the mean scales and components scores (p > 0.05).

**Table 6:** One-way ANOVA comparison of the Hausa SF-12 scales and components scores by age group.

	Age group						
SF-12	18–24	25–44	45–64	≥ 65			
Scale	Mean (SD)	Mean (SD)	Mean (SD)	Mean (SD)	F-ratio	p-value	ηp2
PF	46.0 (20.8)	35.3 (20.2)	36.7 (20.3)	30.5 (21.1)	2.211	0.088	0.03
RP	42.1 (38.2)	26.1 (33.1)	21.1 (30.0)	12.1 (26.2)	3.501	0.016*	0.05
BP	57.9 (30.1)	43.0 (23.1)	43.2 (23.1)	31.4 (25.5)	4.437	0.005*	0.06
GH	50.0 (26.3)	40.7 (22.3)	38.4 (21.9)	27.7 (23.3)	2.729	0.011*	0.06
VT	51.7 (22.4)	42.7 (26.1)	40.2 (25.8)	28.1 (25.5)	3.447	0.018*	0.05
SF	63.1 (34.7)	53.0 (29.4)	51.9 (28.2)	46.2 (29.9)	1.238	0.297	0.01
RE	44.7 (40.4)	30.7 (37.1)	22.4 (31.8)	24.0 (35.0)	2.478	0.063	0.03
MH	56.8 (20.5)	43.2 (23.4)	44.1 (21.2)	38.5 (22.6)	2.708	0.046*	0.04
**Component**							
PCS-12	38.2 (8.33)	34.7 (6.75)	34.4 (6.82)	31.5 (5.68)	3.591	0.015*	0.05
MCS-12	43.5 (11.4)	38.6 (11.0)	39.0 (8.53)	38.8 (10.1)	2.210	0.088	0.03

*p < 0.05; ηp2 indicates partial eta squared

PF, physical functioning; RP, role-physical; BP, bodily pain; GH, general health; VT, vitality; SF, social functioning; RE, role emotional; MH, mental health; PCS-12, physical component summary; MCS-12, mental component summary

**Table 7:** Independent t-test comparison of scales and components score of the Hausa SF-12 by gender and habitation.

	Gender				Habitation			
SF-12	Men	Women			Urban	Rural		
Scale	Mean (SD)	Mean (SD)	t-cal	p-value	Mean (SD)	Mean (SD)	t-cal	p-value
PF	37.6 (19.8)	34.4 (21.8)	1.063	0.289	35.3 (24.4)	37.0 (17.7)	-0.994	0.346
RP	24.3 (22.7)	22.7 (32.9)	0.356	0.722	23.7 (32.7)	23.7 (31.7)	-0.224	0.823
BP	44.7 (40.2)	40.2 (25.0)	1.236	0.218	40.9 (26.4)	44.3 (23.6)	-0.668	0.505
GH	39.4 (23.7)	37.9 (22.4)	0.427	0.670	39.3 (23.7)	38.5 (22.8)	-0.620	0.951
VT	40.3 (24.9)	40.7 (27.7)	-0.119	0.905	41.0 (26.7)	40.1 (25.7)	0.110	0.912
SF	53.8 (29.5)	50.6 (29.7)	0.744	0.458	49.6 (31.4)	54.5 (28.2)	-0.782	0.435
RE	27.2 (35.2)	27.9 (34.8)	-0.135	0.893	28.1 (33.6)	27.0 (36.0)	0.000	1.000
MH	44.6 (22.7)	43.7 (21.9)	0.266	0.790	42.8 (22.0)	45.2 (22.6)	-0.347	0.729
**Component**								
PCS-12	34.9 (7.16)	33.8 (6.58)	1.063	0.289	33.8 (7.51)	34.9 (6.53)	-1.121	0.264
MCS-12	39.2 (10.4)	38.2 (9.79)	0.667	0.505	38.2 (10.1)	39.3 (10.2)	-0.717	0.474

PF, physical functioning; RP, role-physical; BP, bodily pain; GH, general health; VT, vitality; SF, social functioning; RE, role emotional; MH, mental health; PCS-12, physical component summary; MCS-12, mental component summary

#### Internal consistency

Internal consistency (*n =* 200) as measured by Cronbach-α if item deleted was 0.73 for the PCS-12 and 0.78 for the MCS-12.

#### Test-retest reliability

**Fig 2:**
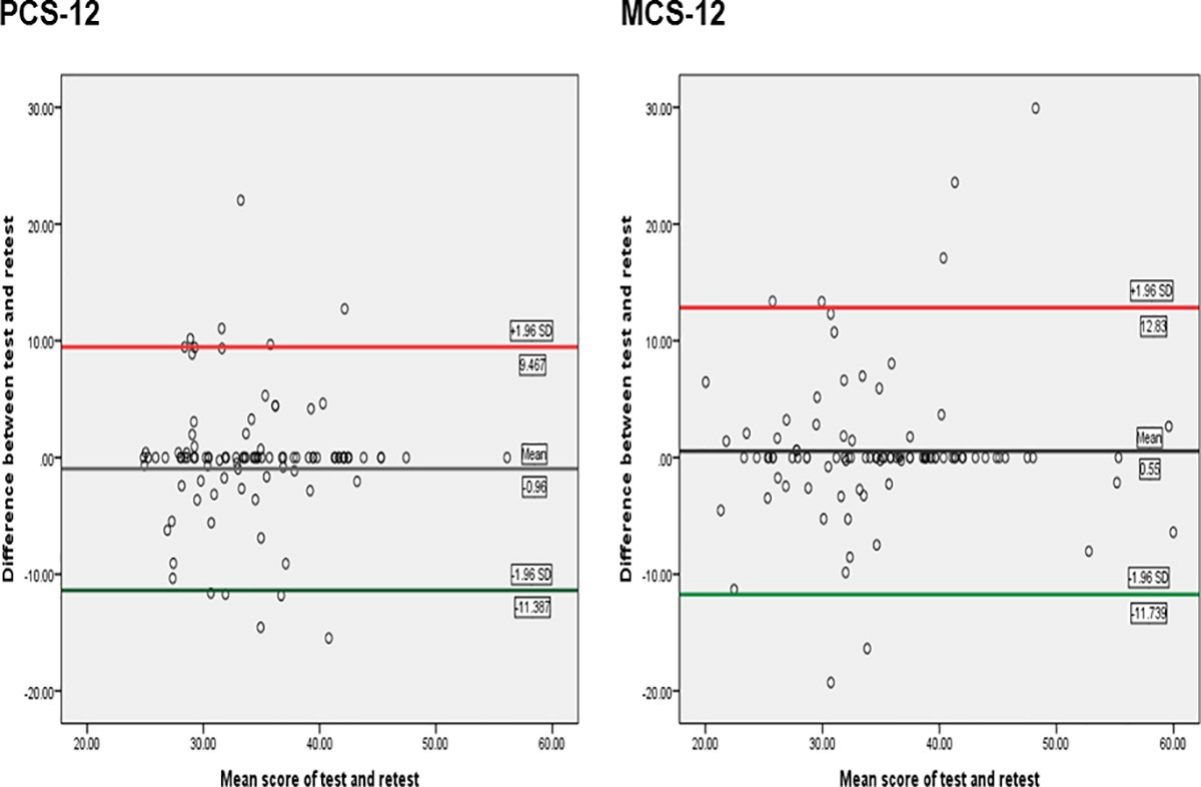
Bland–Altman plot for test-retest agreement of PCS-12 and MCS-12. PCS-12 = physical component summary; MCS-12 = mental component summary.

Test-retest reliability (*n =* 100) as measured by ICC was 0.79 (95% CI: 0.69–0.86) for the PCS-12 and 0.85 (95% CI: 0.77–0.89) for the MCS-12. The Bland–Altman analysis showed a mean difference of –0.96 and 0.55 for PCS-12 and MCS-12 respectively. The limits of agreement for PCS-12 were –11.387 to 9.467 and –11.739 to 12.839 for MCS-12. The results show minimal systematic bias (Fig 2).

## Discussion

With the rising prevalence and burden of chronic conditions such as LBP in both developed and developing countries [1,59], the assessment of HRQOL of affected individuals using validated outcome measures is essential to guide the choice of treatment and evaluate outcomes. To the authors’ knowledge, this is the first study to report on the translation and validation of the Hausa SF-12 in Hausa-speaking LBP population. The results suggest that the instrument has adequate factorial invariance, construct validity, internal consistency, and test-retest reliability in Hausa-speaking patients with chronic LBP.

Cross-cultural adaptation of the Hausa SF-12 was easy and straight forward except for some minor modifications in wordings for items 2 and 11 to ensure familiarization in Hausa culture. The translators ensured that the Hausa SF-12 reached conceptual equivalence to the original English version. The instrument was clear without any difficulty with comprehension of items despite the inclusion of both literates and non-literates as well as urban and rural patients with the goal of having a broader application of the instrument. The response rate was 100% suggesting acceptability of the instrument, even though majority of the respondents were not self-administered. Self-administration of the SF-12 was found to be associated with poor completion rates in a previous validation study [60].

The fact that floor effects were reached in the PF scale suggests that the respondents have limitations in performing physical activities due to chronic LBP. On the other hand, ceiling effects in the GH and VT scales suggest that the respondents perceived somewhat better overall health and energy. The findings that both floor and ceiling effects were reached for the RP and RE scales might suggest that while some respondents have issues with their physical health and emotions due to chronic LBP, others tend to have no issues with their physical health and emotions due to chronic LBP. These findings are inconsistent with those of previous validation studies that found no floor or ceiling effects in the SF-12 among the general population [33–36]. The mean PCS-12 (34.5) and MCS-12 (38.9) scores obtained in our study suggest lower HRQOL compared to the scores of nine countries drawn from the general population [20]. These findings are not surprising given that our subjects were typical sufferers of chronic LBP. It is believed that individuals with chronic LBP experience sub-optimal quality of life due to pain and reduced function [9,10–12].

Regarding the factor structure of the Hausa SF-12, the CFA suggest that modifications in the original conceptual model reflecting physical and mental health measures were necessary to adequately fit the sample variance/covariance matrix. The removal of items 3 and 4 due to redundancy improved model fit of the conceptual SF-12 model. The redundancy of these items might be due to irrelevancy to the sample even though the respondents did not report any problem with the items during the cross-cultural adaptation process. Specifically, item 3 which is concerned with limitation in climbing several flights of stairs seems to be inapplicable to our sample since most people in northwestern Nigeria especially rural dwellers do not usually live in houses with stairs. However, for item 4 which is concerned with problems regarding daily work or physical activities, it can be speculated that responding to the question “accomplished less than you would like” maybe somewhat problematic given that the item response has only 2 options (yes or no), unlike in the reversed version (SF-12v2) where the item response has been extended from 2 to 5 which gives more response categories [61].

Though the AVE values obtained for the PCS-12 and MCS-12 were smaller than the acceptable value of ≥ 0.5 [62], however, according to Fornell and Larcker [46], the convergent validity is still adequate since the corresponding CR values were higher than 0.6. It should be noted, however, that AVE is a strict measure of convergent validity and smaller numbers of scale items result in lower reliability levels as in the case of our refined model [63]. Another possible explanation for the lower AVE values is that the factor loadings, especially those of the PCS component, were mostly less strong (< .70). It has been documented that AVE < 0.5 signifies average item loading less than .70 [62]. Thus, items of the Hausa SF-12 components exhibited more error variance than explained variance. Although, further model remedies may improve the AVE values, however, additional deletion of potential redundant items reveals deterioration of the model fit. In light of the foregoing, the convergent validity and reliability of the Hausa SF-12 are therefore supported.

To the best of our knowledge, this is the first study to examine the factorial or measurement invariance of the SF-12 in population with chronic LBP. Interestingly, the proposed refined model exhibited a good fit in the demographic groups according to age, gender, and habitation. Factorial invariance of the Hausa SF-12 was fully supported evidence by the adequate model fit statistics and indices in terms of configural, metric and scalar invariance analyses. These findings suggest the ability of the Hausa SF-12 to perform similarly well among younger adults and adults, men and women, as well as urban and rural populations with chronic LBP. Our findings are in concordance with that reported by Galenkamp et al [50] who found evidence of factorial invariance of the SF-12 for different demographic variables including age and gender among a multi-ethnic sample (HELIUS) of over 23,000 participants in Netherland. Even though some previous studies [64,65] showed a violation of the assumption of factorial invariance pertaining to age and gender, such violation (differential item functioning) did not translate into significant changes in the pattern of SF-12 components scores across these variables. For habitation, no prior publication could be found in the literature examining factorial invariance of the SF-12 across this particular variable. Our study, therefore, provides a piece of evidence for the SF-12 to perform well among urban and rural populations with chronic LBP.

Results of the construct validity of the Hausa SF-12 were very encouraging as the *a priori* hypotheses were confirmed for the convergent (11:12; 91% success rate) and divergent (10:12; 83% success rate) validity. Convergent validity was demonstrated by the higher correlations of items with own hypothesized component whereas divergent validity was demonstrated by the lower correlations of items with component less in common. However, item 5 (RP2) and 8 (BP) which supposed to correlate higher with the PCS-12, also had a relatively high correlation with the MCS-12. This is somewhat similar to the findings obtained for the original English SF-12 version reported by Ware et al [19] where the VT, GH, and SF scales had a relatively high correlation with both the PCS-12 and MCS-12. Other validations such as the Iranian [34], Tunisian [35], and Moroccan [36], however, did not report such kind of correlation pattern. The result that the PCS-12 and MCS-12 were weakly correlated in our study, also confirmed the discriminant ability of the instrument.

Known-groups validity of the Hausa SF-12 was supported in terms of its ability to differentiate between subgroups of respondents who differed in age but in gender and habitation. Older respondents were found to exhibit poor health in the scores RP, BP, GH, VT, and MH scales, as well as PCS-12, compared to younger respondents. These findings are consistent with the results of previous studies on general population [33,34]. Though no significant difference was reached for the scores of PF, SF, and RE scales, as well as MCS-12 across the different age groups, there appears to be a trend suggesting a decrease in scores of these variables among older respondents. The findings that the SF-12 scales and summary scores were unable to distinguish between subgroups of respondents on the basis of gender and habitation might be attributed to the respondents’ specific condition (i.e. chronic LBP). Thus, it can be inferred that men and women, as well as urban and rural respondents, are equally affected by chronic LBP. On this basis, our findings should therefore be interpreted with caution.

Internal consistency for the PCS-12 (0.78) and MCS-12 (0.79) lies within the recommended Cronbach-α range of 0.70–0.95 [41], thus indicating adequate reliability. These findings correspond with that obtained for the original English version (PCS-12 = 0.77; MCS-12 = 0.80) in patients with LBP [21] and also among other language versions [34,36]. In a similar fashion, the calculated ICC for the PCS-12 (0.79) and MCS-12 (0.85) were adequate suggesting good test-retest reliability. Ware and colleagues [39], however, reported higher ICC (0.89) for the PCS-12 but slightly lower (0.75) for MCS-12 when compared to our findings. In contrast, lower ICC values for the PCS-12 (0.47) and MCS-12 (0.72) were reported for the Brazilian version in patients with progressive systemic sclerosis [66]. The variations in the values of ICC across studies can be a result of variations in the population sampled, methods of assessments and intervals between assessments. Because ICC does not take into account the size of measurement error that is clinically relevant, Bland–Altman plots were also performed to assess limits of agreement of the Hausa SF-12. The results showed minimal systematic bias as the mean difference for both the PCS-12 (-0.96) and MCS-12 (0.55) was close to zero with few outliers and most points lie within the 95% limits of agreement. Overall, the reliability results of the present study suggest that the Hausa SF-12 is a reliable measure of health status.

This study is not without potential limitations which should be considered when interpreting the results. Firstly, though our translation process followed the IQOLA protocol, few optional steps such as rating of the difficulty and quality of the forward and backward translations were skipped due to lack of funding and limited resources. Secondly, the study included only patients with chronic LBP; thus, the study results may not be generalized. It should be noted that clinimetric properties of a measure are influenced by population characteristics and so can change in different population groups [41,67]. Furthermore, responsiveness which aims to measure change over time was not conducted. Subsequent studies should therefore consider establishing stronger psychometric properties of the Hausa SF-12 in general population and other patient groups.

## Conclusions

The results of this study suggest that the Hausa SF-12 was successfully developed and showed evidence of factorial invariance across age, gender, and habitation. The construct validity, internal consistency, and test-retest reliability were satisfactory. However, stronger psychometric properties need to be established in general population and other patients in future studies. The instrument proved to be useful for clinical and research purposes in Hausa-speaking patients with chronic LBP. It may also support the uptake of multicentric and multinational studies such as the global health initiatives which usually involve concurrent research activities in culturally and linguistically diverse countries.

## Supporting information

S1 Appendix Hausa version of SF-12. (PDF)

S1 Data Hausa SF-12 data. (XLSX)
