# Supplementary material for: The Hausa 12-item short-form health survey (SF-12): Translation, cross-cultural adaptation and validation in mixed urban and rural Nigerian populations with chronic low back pain
Source: PLoS One. 2020 May 7;15(5):e0232223. doi: 10.1371/journal.pone.0232223 (PMC7205304; doi:10.1371/journal.pone.0232223)
Supplement: S1 Appendix — (PDF) [file pone.0232223.s001.pdf]

SF-12 Bincike Game da Lafiya

Wannan bincike ne da ke son jin ta bakinka game da lafiyarka. Waɗannan bayanai za su taimaka wajen adana bayanan yadda ka ke ji ajikinka da kuma yadda ka ke aiwatar da ayyukanka na yau da kullum. **Amsa kowace tambaya ta hanyar zaɓar amsa guda ɗaya tak.** Idan ba ka da tabbacin yadda za ka amsa wata tambaya, ana son ka ba da amsar da ta dace da kanka daidai fahimtarka.

1. **A faɗaɗe dai, shin za ka iya cewa lafiyarka tana da**:

☐1 Cikakken inganci ☐2 Inganci ƙwaran gaske ☐3 Inganci ƙwarai ☐4 Inganci ba laifi ☐5 Rashin inganci

Waɗannan tambayoyi da ke biye sun shafi irin ayyukan da za ka iya aiwatarwa ne a cikakken wuni. Shin Ingancin lafiyarka ya kawo maka nakasu wajen aiwatar da waɗannan ayyuka ? idan haka ne, to sau nawa ne?

	**Ee**<br>**Ya kawo min naƙasu sau da yawa**	**Ee**<br>**Ya kawo min naƙasu amma kaɗan**	**A'a**<br>**Bai kawo min naƙasu ba**
2. **Matsakaitan** ayyuka kamar matsar da teburi ko ɗaukar kwandon shara ko wasan kwari da baka.	☐1	☐2	☐3
3. Hawan matattakalar bene **da yawa**.	☐1	☐2	☐3

A cikin sati huɗu baya shin ka ci karo da ɗayan waɗannan matsaloli game da aikinka ko sauran al'amuranka na yau da kullum a sakamakon lafiyar jikinka?

	**Ee**	**A'a**
4. **Kammala ayyukan ƙasa** da yadda ka ke buƙata.	☐1	☐2
5. Na samu ƙarancin yin aikina da sauran ayyukana.	☐1	☐2

A cikin sati huɗu baya, shin ka ci karo da ɗayan waɗannan matsaloli game da aikinka ko sauran al'amuranka na yau da kullum a dalilin matsalar tunani (kamar damuwa ko zaƙuwa)?

	**Ee**	**A'a**
6. **Kammala ayyukan ƙasa** da yadda ka ke buƙata.	☐1	☐2
7. Rashin mayar da hankali wajen aiwatar da ayyukan **saɓanin yadda aka saba**	☐1	☐2

8. **A cikin sati huɗu baya, sau nawa ne raɗaɗin ciwo ya kawo maka cikas wajen ayyukanka na yau da kullum (wannan ya haɗar da ayyukanka na gida da na waje)?**

☐1 Sam ☐2 Kaɗan ne ☐3 Jefi-jefi ☐4 Kaɗan ne matuƙa ☐5 Sosai

Waɗannan tambayoyi sun shafi yadda ka/ki ke ji a sati huɗu baya.
Wajen amsa kowace tambaya, ana son ka ba da amsar da ta yi kusa da yadda ka ke ji.

A waɗanne lokuta ne kuma sau nawa ne cikin sati huɗu da suka gabata…

	Kowane lokaci	Yawancin lokuta	Mafi yawan lokuta	A wasu lokutan	A wasu yan lokutan	Ba kowane lokaci ba
9. Shin ka samu kwanciyar hankali da nutsuwa?	☐1	☐2	☐3	☐4	☐5	☐6
10. Shin ka na da kuzari sosai?	☐1	☐2	☐3	☐4	☐5	☐6
11. Shin ka samu damuwa da karayar zuciya?	☐1	☐2	☐3	☐4	☐5	☐6

12. **A tsawon sati huɗu baya, sau nawa ne kuma a waɗanne lokuta matsalar rashin lafiyar jikinka da ta tunani ta kawo cikas ga al'amuranka na rayuwa (kamar ziyartar abokanka da 'yan'uwa da sauransu.)?**

☐1 Kowane lokaci ☐2 Yawancin lokuta ☐3 Mafi yawan lokuta ☐4 A wasu lokutan ☐5 Ba kowane lokaci ba
